# Factors to Describe the Outcome Characteristics of a CI Recipient

**DOI:** 10.3390/jcm13154436

**Published:** 2024-07-29

**Authors:** Matthias Hey, Kevyn Kogel, Jan Dambon, Alexander Mewes, Tim Jürgens, Thomas Hocke

**Affiliations:** 1 ENT Clinic, UKSH Kiel, 24105 Kiel, Germany; kevyn.kogel@uksh.de (K.K.); janandreas.dambon@uksh.de (J.D.); stu226070@mail.uni-kiel.de (A.M.); 2 Institute of Acoustics, University of Applied Sciences Lübeck, 23562 Lübeck, Germany; tim.juergens@th-luebeck.de; 3 Cochlear Deutschland, 30539 Hannover, Germany; thocke@cochlear.com

**Keywords:** cochlear implant, speech recognition, signal processing, speech audiometry

## Abstract

**Background:** In cochlear implant (CI) treatment, there is a large variability in outcome. The aim of our study was to identify the independent audiometric measures that are most directly relevant for describing this variability in outcome characteristics of CI recipients. An extended audiometric test battery was used with selected adult patients in order to characterize the full range of CI outcomes. **Methods**: CI users were recruited for this study on the basis of their postoperative results and divided into three groups: low (1st quartile), moderate (medium decentile), and high hearing performance (4th quartile). Speech recognition was measured in quiet by using (i) monosyllabic words (40–80 dB SPL), (ii) speech reception threshold (SRT) for numbers, and (iii) the German matrix test in noise. In order to reconstruct demanding everyday listening situations in the clinic, the temporal characteristics of the background noise and the spatial arrangements of the signal sources were varied for tests in noise. In addition, a survey was conducted using the Speech, Spatial, and Qualities (SSQ) questionnaire and the Listening Effort (LE) questionnaire. **Results**: Fifteen subjects per group were examined (total N = 45), who did not differ significantly in terms of age, time after CI surgery, or CI use behavior. The groups differed mainly in the results of speech audiometry. For speech recognition, significant differences were found between the three groups for the monosyllabic tests in quiet and for the sentences in stationary (S0°N0°) and fluctuating (S0°NCI) noise. Word comprehension and sentence comprehension in quiet were both strongly correlated with the SRT in noise. This observation was also confirmed by a factor analysis. No significant differences were found between the three groups for the SSQ questionnaire and the LE questionnaire results. The results of the factor analysis indicate that speech recognition in noise provides information highly comparable to information from speech intelligibility in quiet. **Conclusions**: The factor analysis highlighted three components describing the postoperative outcome of CI patients. These were (i) the audiometrically measured supra-threshold speech recognition and (ii) near-threshold audibility, as well as (iii) the subjective assessment of the relationship to real life as determined by the questionnaires. These parameters appear well suited to setting up a framework for a test battery to assess CI outcomes.

## 1. Introduction

Cochlear implants (CIs) represent an option for patients with profound hearing loss [1,2,3] or with insufficient residual speech recognition [4,5] and today, in some countries, even for asymmetrical hearing loss of various degrees [6].

At the very beginning of CI provision, the observed postoperative word recognition scores (WRS) were rather low because most patients had long-standing and high-degree hearing loss before the implantation [7]. CI candidacy has changed from the beginning of clinical cochlear implant care [2,7,8,9,10] to nowadays, where the vast majority of CI recipients show improved speech perception after CI provision [11,12,13,14,15,16], as up to two-thirds of the patients have a preoperative residual speech recognition [17,18].

As soon as larger populations were investigated, the enigma of (unexplained) poor performance [19,20] became evident, and it still persists [17,18]. Several attempts have been made to identify predictive factors for WRS after CI provision [12,14,19,21,22]. Those studies focussed on the relationships between various preoperative and postoperative measurements in order to predict the results of the latter ones. Supra-threshold presentation of monosyllabic words at 65 dB in quiet was mainly considered [14,19,21,23].

For the postoperative assessment of implantable hearing systems, a large number of measures may be feasible [4,24,25,26]. Supra-threshold measures of monosyllabic words at the conversational presentation level are widely used [12,19,21,24]. Everyday listening situations include speech in quiet as well in noise [27,28,29]. Recent data logging studies with speech processors indicate that low-level speech is also present to a substantial extent [30,31,32]. Audiometric surrogate parameters for low-level speech include word or sentence recognition scores at 40 and/or 50 dB SPL and speech reception thresholds (SRT) of numbers [24,33,34,35,36].

An aspect that has more recently been a focus in speech audiometry research is the increased focus on ecological validity. As a result, tests using sentences in noise are nowadays also obligatory in aftercare [6]. Matrix tests using different spatial loudspeaker configurations and competing signal conditions are in wide use [37,38,39,40].

Complementing the audiometric assessment, patient-reported outcome measures have become an established tool in the aftercare of recipients of implanted hearing systems. Numerous questionnaires, visual analog scales, and ecological momentary assessments [41,42] are available. These have been adapted to the special requirements of patients with hearing systems.

The availability of so many audiological methods raises the question of which ones should be chosen for use in routine clinical audiometry. Together, all these measures represent a statistically overdetermined system, an assertion that is supported by the many correlations found among the various outcome measures [24,43,44]. Two aspects are worth considering in view of such overdetermination: (i) the burden of time and concentration for the patient limits the daily available time frame; (ii) clinical resources should be allocated with maximum efficiency.

The aim of this study was to identify the most relevant measures, specifically those that provide information independent of other measures, for the purpose of describing as completely as possible the outcome characteristics of CI recipients. It was not the aim of this study to investigate the causes of the poor performance, but to describe the reliable audiological assessment of this performance with regard to the available methods.

Therefore, a test battery based on Hoth and Müller-Deile [24] was extended to measure numerous different outcome parameters in CI patients. The test battery consisted of speech recognition in quiet for numbers and the performance intensity function of words in quiet, which are known to provide useful information for postoperative rehabilitation, system fitting [24,34,36,45], and its considerable importance for everyday life as derived from data logging studies [28,32]. Furthermore, sentences in quiet, sentences in noise with different kinds of noise and different loudspeaker configurations, and questionnaires for subjective patient feedback on speech recognition, directional hearing, hearing quality, and listening effort were included in the test battery.

This test battery was investigated with patients representing the whole range of best, moderately, and poorly performing patients, following the classification proposed by Rieck et al. [23]. An exploratory principal component analysis (PCA) was used to investigate the variability within the three groups with respect to this test battery. Since the portion of poorly performing patients is relatively low [11,18,21,46] to allow for a PCA, all poor-performing subjects had to be included. Afterward, the resulting group size was matched by the moderately and best-performing groups.

## 2. Materials and Methods

### 2.1. Research Participants and Classification

Forty-five participants were recruited for this study, which was approved and conducted in accordance with local university ethics approval (study ID 444/23; 23 February 2023). All procedures involving human participants were performed in accordance with the ethical standards of the institutional and national research committee, as well as the 1964 Helsinki Declaration and its later amendments or comparable ethical standards.

All patients were adults with post-lingual onset of deafness. They had received Nucleus cochlear implants (Cochlear Limited, Macquarie Park, NSW, Australia) and had been using them for at least one year [12,47,48]. They were recruited from our clinical database, which included 538 individuals [23].

The patients were classified on the basis of their performance in the Freiburg monosyllabic test at 70 dB SPL conducted two years (instead of one case at 1 year) after receiving the CIs.

Those who scored above the third quartile were considered to have good speech recognition performance and were termed ‘high performers’ (HP). Patients who scored below the first quartile were termed ‘low performers’ (LP). Patients in the median range (45th to 55th percentile) were referred to as ‘moderate performers’ (MP). The classification was performed monaurally and excluded patients who received binaural treatment with different outcome levels (one side HP and one side LP) as well as CI recipients with normal hearing in the other ear.

### 2.2. Audiometric Test Procedures

The tests were conducted in a sound-shielded audiometric test booth [49] using calibrated loudspeakers placed 1 m away from the patient. Participants with bilateral or bimodal hearing were tested on one ear with the contralateral sound processor switched off and the non-tested ear masked if necessary. If both ears were in the same performance group, patients were allowed to choose which ear to use.

All speech comprehension tests were presented through a computer-based audiometer (Equinox; Interacoustics, Middelfart, Denmark with evidENT 3 software, Merz Medizintechnik, Reutlingen, Germany). For speech in quiet, the Freiburg monosyllabic words were applied frontally [50] at presentation levels of 40, 50, 65, and 80 dB SPL. Items from each list were presented in randomized order to minimize any repetitive learning effect. Additionally, the SRT for Freiburg two-digit numbers was measured. The “percent correct” score of a measured list of 10 numbers (greater or smaller than 50%) was used to increase or decrease the presentation level in 5 dB steps for the subsequent list. The SRT was determined by interpolation.

For speech recognition in noise, the Oldenburg sentences (a German version of a Matrix test) were used [37], containing 30 sentences for each list. An adaptive procedure [51] was applied aiming to determine the SRT (the signal-to-noise ratio (SNR) yielding a 50% words correct score). All of the CI users in this study were accustomed to this adaptive test procedure. To reduce the procedural learning effect with the Oldenburg sentence test [52], training was conducted (one list of 30 sentences) before each session. Afterward, the words correct score for the Oldenburg sentences in quiet was measured at 70 dB SPL using 30 sentences. For investigation in noise, according to Hey et al. [52], only recipients with an SRT in S_0_N_0_ (speech and stationary Oldenburg noise from front) better than 5 dB SNR were included. If a patient was unable to meet this criterion, the SRT was set to 5 dB SNR for further analysis. In addition to this measurement, which is the widely used quasi-standard for measurements in noise [53,54], we used a setup aiming for more ecological validity. This implemented the Oldenburg sentences with fluctuating Icra5 noise [55] and separated signal sources: speech coming from the front and the competing signal coming from the side of the CI (S_0_N_CI_) [56].

### 2.3. Questionnaires

To obtain individual subjective feedback on the sound quality obtained with the CI sound processors, the German version of the SSQ questionnaire [57,58] was used, which is known to show a high test–retest accuracy [59]. Additionally, the listening effort questionnaire [60] was used on the same day as speech testing.

Hearing quality in the patients’ everyday lives was determined by using the short version of the German SSQ questionnaire [58]. This questionnaire contains a total of 17 items for the categories of speech recognition, directional hearing, and hearing quality. The rating scale for the SSQ questionnaire items ranges from not at all (0) to perfect (10). A high scale value corresponds to a good assessment of the corresponding hearing situation.

To rate the listening effort of CI patients, the questionnaire of Schulte et al. [58] was chosen. This questionnaire focuses on the categories ‘understanding in noise’, ‘understanding with impaired signal quality’, and ‘understanding in quiet and with lip-reading’, with a total of 17 questions. It shows results on a rating scale ranging from not stressful (0) to extremely stressful (10); thus, a high scale value corresponds to a poor assessment of the corresponding hearing situation.

### 2.4. Analysis

Data are presented as boxplots. Each boxplot shows the median (solid center line), the 25th and 75th percentiles (box limits), and the 5th and 95th percentiles (whiskers) on the left, with individual scores indicated on the right. The Kruskal–Wallis test was used for group comparison. Subsequent post hoc analyses were carried out using the Wilcoxon rank sum test.

The PCA was carried out using the maximum likelihood method. The analysis of the data using the multivariate Henze–Zirkler test for normal distribution showed that a multivariate normal distribution can be assumed, which is a prerequisite for this analysis method [61]. Analysis was performed using Matlab (Mathworks Inc., Natick, MA, USA). If a patient was unable to perform the sentence test adequately in stationary noise, this SRT was set to +5 dB SNR in order to take account of missing data [52]. A comparable procedure was used for the sentence test in fluctuating background noise, where the SRT was set to 10 dB SNR if a value was missing.

## 3. Results

A total of 45 CI patients were recruited for the study. They were assigned to three groups: LP, MP, and HP. The study measurements for speech audiometry in quiet and the questionnaires were successfully completed for all users examined except one LP subject who did not do the questionnaire. Table 1 provides further information about the participants. The mean age of the subjects in the LP group was noticeably lower than in the HP and MP groups, and the mean time since CI implantation was shorter in the MP group than in the HP and LP groups. The usage time of CIs differs only slightly between the groups. However, these differences for age, time after CI surgery, and usage time of CI per day were not statistically significant (Kruskal–Wallis test; χ^2^ = 5.14, *p* = 0.08; χ^2^ = 0.85, 0.66 and χ^2^ = 0.82, *p* = 0.66, respectively) and are not considered likely to have influenced the study’s result.

**Figure 1:**
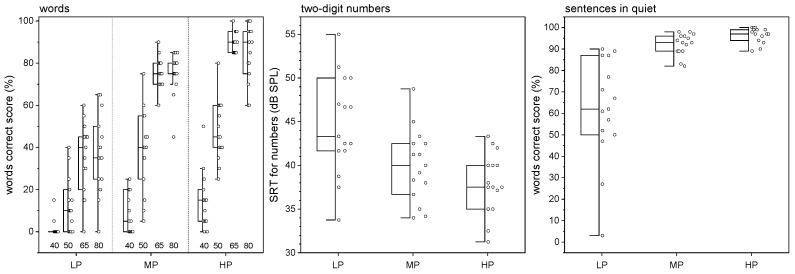
Speech recognition in quiet is presented as boxplots. (**Left**) Grouped data of words correct score for Freiburg monosyllabic words depending on presentation level. (**Middle**) SRT for two-digit numbers. (**Right**) Words correct score for Oldenburg sentences. Groups are assigned as follows: LP—low performer, MP—moderate performer, and HP—high performer.

The results for speech recognition for the Freiburg WRS are shown as box plots depending on stimulation level for the three patient groups examined in Figure 1. The grouping of the patients was based on postoperative speech recognition with CIs [23]. All patients confirmed their allocation to the LP, MP, and HP groups in the Freiburg word test [62] according to its test–retest accuracy. Patients of the low-performing group showed higher variability in speech test data, as the first quartile of the performance includes monosyllabic words correct score from 0% up to 55%. 

For a presentation level of 80 dB SPL, the median speech recognition was 90% for the HP group, 75% for the MP group, and 35% for the LP group. The Kruskal–Wallis test revealed significant differences between the groups, as confirmed by the Wilcoxon rank sum test in a post hoc analysis for HP–LP (*p* < 0.001, zvalue = 4.45) and MP–LP (*p* < 0.001, zvalue = −4.44) pairs and, less clearly significant, HP–MP (*p* = 0.03, zvalue = 2.12). At 65 dB SPL, the median speech recognition for the HP, MP, and LP groups was 90%, 75%, and 40%, respectively. Significant differences between the groups were also found at this level with the Kruskal–Wallis test, and the Wilcoxon rank sum test confirmed these differences between all groups with *p* < 0.001 (HP-LP zvalue = 4.68, HP-MP zvalue = 4.23, MP-LP = −4.65). At 50 dB SPL, the Kruskal–Wallis test also showed significant differences between the groups, which were confirmed by the Wilcoxon rank sum test between HP and LP (*p* < 0.001, zvalue = 4.36) and MP and LP (*p* < 0.001, zvalue = −3.46); however, the HP and MP groups (*p* = 0.16, zvalue = 1.42) did not differ significantly at this SPL level. The measurement results at 40 dB SPL showed significant differences in speech recognition between the three groups. However, the post hoc analysis showed a significant difference only between HP and LP (*p* < 0.001, zvalue = 3.83).

The results of the SRT for Freiburg numbers are shown in Figure 1 (middle). The Kruskal–Wallis test confirms a significant difference between the three groups (χ^2^ = 11.11, *p* < 0.01) and the following post hoc analysis HP–LP (*p* < 0.01, zvalue = −3.12) and MP–LP (*p* = 0.03, zvalue = 2.22).

The results of the Oldenburg sentences in quiet are shown in Figure 1 on the right. The Kruskal–Wallis test showed that there were significant differences between the three groups.

**Figure 2:**
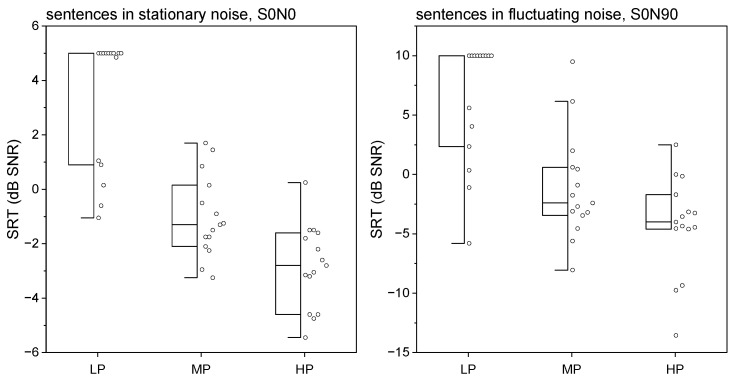
Speech recognition in noise presented as boxplots. (**Left**) SRT for the Oldenburg sentences in stationary noise; speech and noise from the front. (**Right**) SRT for the Oldenburg sentences in fluctuating noise; speech from the front and noise from the side of the CI.

Figure 2 (left) shows the results of the SRT for the Oldenburg sentences in stationary noise and frontal presentation of speech and noise as boxplots. The median SRT for the HP group was –2.8 dB SNR, with a range of 0.25 to –5.45 dB SNR. Figure 2 (right), in contrast, shows speech recognition in fluctuating noise with spatially separated signal sources. The HP and MP groups revealed no significant difference (*p* = 0.06, zvalue = −1.85).

**Figure 3:**
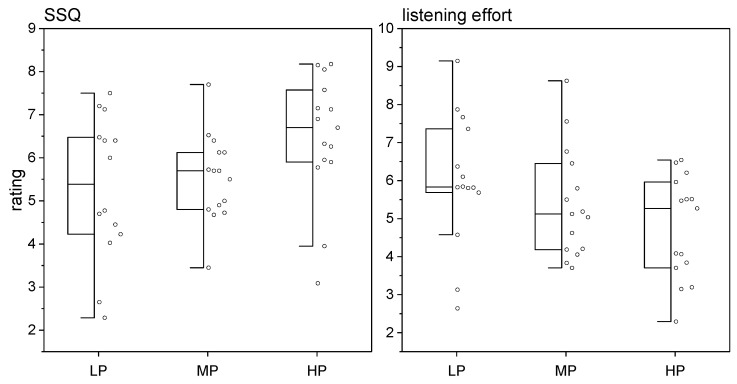
Results of the questionnaires for the three sub-groups as boxplots. (**Left**) SSQ questionnaire. A high SSQ value corresponds to a positive evaluation for the situations surveyed. (**Right**) Listening effort questionnaire. A high value on the listening effort questionnaire scale corresponds to a negative evaluation for the situations surveyed.

The results of the patients’ subjective feedback using two questionnaires are shown in Figure 3. No significant differences were found between the performance groups. It should be added that the performance groups did not differ significantly in the SSQ subdomains (Kruskal–Wallis for speech understanding χ^2^ = 3.3; *p* = 0.20, for spatial orientation χ^2^ = 2.8; *p* = 0.25, listening quality χ^2^ = 3.3; *p* = 0.19, and listening effort χ^2^ = 5.3; *p* = 0.07).

A PCA was carried out using the maximum likelihood method. The analysis of the data with the multivariate Henze–Zirkler test for normal distribution showed that a multivariate normal distribution (*p* = 0.21, HZ lognormal variance = 0.02, HZ statistic = 0.98) can be assumed, which is a prerequisite for this analysis method [61]. The Kaiser–Meyer–Olkin measure for determining the intercorrelation between the variables was 0.87. According to Klopp [61], this corresponds to a good suitability of the data for a factor analysis. The results of the factor analysis are shown in Table 2. The analysis of the eigenvalues revealed that no more than three factors should be selected for calculation, as the eigenvalue for the fourth factor is clearly below one at 0.51. This indicates that most of the observed variances can be well explained by the first three factors. Analysis of the eigenvalues showed that 84% of the variability is explained by the first three components, with 60% for the 1st, 14% for the 2nd, and 10% for the 3rd component.

Results for supra-threshold speech audiometry (WRS at 65 and 80 dB SPL, sentences in quiet as well as in stationary and fluctuating noise) show high absolute value loadings on factor 1, with the highest value for monosyllabic words at 80 dB SPL. Near-threshold speech tests (numbers, words at 40 and 50 dB SPL) are dominant for the 2nd factor. The questionnaires show a clear loading for the 3rd factor; this applies in particular to the SSQ.

## 4. Discussion

In our investigation, we measured audiometric and subjective outcome parameters for a group of 45 experienced CI patients. To address the issue of poor performance, we weighted the share of poor performers equally with the shares of excellent and medium performers, allowing reliable differentiation between those groups.

The basic audiometric characteristics of a CI recipient can be described by three main components: (1) *Supra-threshold speech recognition*, (2) near-threshold *audibility*, and the subjectively perceived benefit referred to as (3) *patient-reported outcome*. Most remarkably, speech recognition in noise did not load a separate component but was the same as supra-threshold speech recognition in quiet.

All patients in the HP and MP groups were able to perform all audiometric and subjective outcome tests of the complete test battery, unlike some patients in the LP group, in which nine persons were unable to perform the two sentences tests in noise. Their correct score in noise was too low to allow the performance of the adaptive procedure [52]. However, responses for patient-reported outcome measures and ‘numbers in quiet’ were adequate, as all patients were able to respond. For monosyllabic words at different test levels, the measurement procedure could be performed for all patients, although some of the recipients showed a score of ‘no words correct’. Nevertheless, for this test, it was an adequate measure in the LP group, and this result corresponds well with the overall performance of such recipients, as has also been described elsewhere [18].

Browning et al. [63] stated that good speech comprehension can be achieved in different ways. The way in which this is achieved is not the focus of the present study, which primarily concerns an adequate description of the outcome in relation to speech recognition, which is the main goal of CI therapy for high degrees of hearing loss.

According to the Kaiser–Meyer–Olkin value of 0.86, the data collection of this test battery provides a good basis for PCA and the further interpretation presented here.

### 4.1. 1st Component—Supra-Threshold Speech Recognition

The following considerations arise in the interpretation: The first component is described by supra-threshold comprehension. This applies to word comprehension at 65 and 80 dB SPL as well as to the sentence tests in quiet and in noise, which were also carried out at a presentation level of 65 dB SPL. The highest loading of the first component was observed for the monosyllabic word test at 65 and 80 dB SPL and sentences in quiet and noise at 65 dB SPL. On the basis of the present findings and the other investigations [64,65], we would have expected a separate component for comprehension in noise. Surprisingly, to account for variability, speech recognition in noise does load on the same component as conversational level speech in quiet. Weissgerber et al. [46] found that age is the only predicting factor for SRT in noise in a selected group of CI patients with a preoperative maximum WRS greater than 0%. In the present study, the three groups did not differ significantly in age. This may explain the fact that speech in noise did not contribute additional information in our PCA.

### 4.2. 2nd Component—Audibility

Instead of speech recognition in noise, the second component is loaded by monosyllabic WRSs and multisyllabic numbers at lower levels of speech at 50 dB SPL and below. This can be interpreted as audibility [34,45]. The highest representation in this second component is WRS at 50 dB SPL. However, we would recommend the SRT for numbers or an equivalent measure, as this is free of floor effects, while the WRS at low stimulation level yielded in a very large portion of the LP a value of 0%. Additionally, the assessment of the SRT is less frustrating for the CI users while still yielding the information contained in the 2nd component. Furthermore, the numbers are highly redundant. Consequently, the number test represents more audibility and less lexical effects than the monosyllabic test material. The correlation between all near-threshold scores should not be misinterpreted as an argument for omitting assessment of the discrimination function: in this study, we included patients who, in our opinion, were well aided (fitted) [24]. They had completed postoperative rehabilitation and showed stable fitting of their speech processor. However, in order to identify any fitting issues, a mismatch between scores at different levels within the discrimination function of monosyllables can give valuable hints for optimizing the speech processor settings [24,34,36]. Additionally, WRS at medium and even low levels is important for everyday communication [32].

### 4.3. 3rd Component—Patient-Reported Outcome

The third component is loaded by the patient’s perspective on everyday life as assessed by questionnaires. This component is loaded most strongly by SSQ and to a much lesser degree by listening effort. For the German AWMF guideline [53], this is taken into account by the recommended use of the Nijmegen Cochlear Implantation Questionnaire (NCIQ) [66]. The 3rd component can be considered a valuable addition in postoperative quality assurance. Patient-reported outcome measures represent an important part of the outcome measure, but one cannot completely determine the outcome by questionnaires. It has to be mentioned that audiometric measures are not a reliable predictor of patient-reported outcomes in CI patients [67]. On the other hand, audiometric measures of performance prior to CI surgery do not show a reliable correlation to postoperative quality-of-life scores [68]. In the 3rd component, the three groups are hardly distinguishable (see below and in the paragraph Limitations).

Nevertheless, the measure explains the additional variability of 10%. The different preoperative baselines of the individual recipients may provide a rationale for this finding. It is reasonable to assume that a recipient with poor preoperative audiological condition may perceive a great subjective benefit even if his/her WRS is poor compared with that of other recipients [69]. Consequently, the smaller share of variability explained by the third component should not be misinterpreted as implying that subjective rating is only of low importance.

### 4.4. Limitations of the Study

Study participants were grouped according to their former WRS, as this score is the audiometric hub for all therapeutic decisions according to the AWMF guidelines. Consequently, this measure loads the 1st component strongly.

A limited set of audiometric procedures was applied in this study. There is a variety of other German-language tests, such as the following tests in quiet and in noise: the Sotscheck test, the Göttingen and HSM sentences tests, and the digit-triplet test. Furthermore, more spatial settings—as used in recent studies [13,35,40,70]—were not included here. The subset of possible audiometric tests was selected by consideration of the limited time available for concentrated testing of the patients.

The present study did not analyze objective procedures such as electrode impedances, or electrophysiological [71], anatomical [11], and mapping parameters [34,36,45,64]. The focus here was not on fitting but on describing independent determinants of outcome. These additional variables provide the basis for the subject of further, currently ongoing studies.

The selection of patients for this study was based on speech recognition as the dominant target parameter, as described in the AWMF guidelines [53] and various earlier studies [12,19,72]. The audiometric test procedures used separated the LP, MP, and HP groups well. However, this separation was not recognized for patient-reported outcome measures. This may have been due to the fact that other aspects are tested here. For example, the listening effort for HP patients can be high, as they may be integrated into a normal acoustic listening environment through their occupation and are exposed to more background noise than LP patients.

There are also other approaches for defining poor performance in characterizing CI outcomes. In the present study, absolute postoperative performance was used. Another way would have been to consider postoperative performance in comparison with preoperative auditory status—i.e., relative performance, as described by Hoppe et al. [18,23], according to whom specifications for poor performance are hard to meet because they apply to only very few patients of the total collective.

However, the aim here was not to describe the treatment of the LP but only to characterize it. This tool can now be used for patient collectives at other centers and with different CI systems. This test battery might be used for further evaluation of poor-performing patients. This topic of cases with unexpectedly poor speech perception was motivated by Moberly [20] and was described in more detail with respect to speech recognition by Hoppe et al. [18].

## 5. Conclusions

Within the outcome-measure framework for CI patients used here in relation to the specific research or clinical question, a proposal for a test battery for evaluating the therapeutic success of a CI was developed. The three components of audiometrically measured supra-threshold comprehension and audibility, as well as subjective rating of relationship to real life through the questionnaires, are well suited for such a framework. 

Results of the study suggest the use of (i) monosyllabic words at 65 and/or 80 dB SPL, (ii) monosyllabic words at 50 dB SPL and/or SRT of numbers, and (iii) the SSQ questionnaire as a minimum test inventory.

## Figures and Tables

**Table 1:** Patient details. For age, duration of fitting, and use time per day, mean values and standard deviations are given. The side in hearing solution characterizes the tested ear. Groups are assigned as follows: LP—low performer, MP—moderate performer, and HP—low performer.

	Study Population	LP Group	MP Group	HP Group
Group Size	45	15	15	15
Age (years)	61.4 ± 12.6	55.7 ± 12.9	63.7 ± 14.0	64.8 ± 9.3
Time after CI surgery (years)	6.2 ± 4.1	7.0 ± 5.3	4.8 ± 1.6	6.9 ± 4.3
Use time of CI per day (h)	13.7 ± 3.0	13.0 ± 3.7	13.4 ± 2.5	14.7 ± 2.7
**Hearing solution**				
binaural (right)	14	4	5	5
binaural (left)	9	4	1	4
bimodal (right)	14	5	5	4
bimodal (left)	4	0	2	2
monaural (right)	2	1	1	0
monaural (left)	2	1	1	0
**Speech processor**				
CP1100	2	1	0	1
CP1000	37	13	13	11
CP1000 Hybrid	2	0	0	2
CP910	3	1	2	0
CP910 Hybrid	1	0	0	1
**Cochlear implant**				
CIx32	33	9	14	10
CIx12	4	2	0	2
CI24RE(CA)	8	4	1	3

**Table 2:** PCA after Varimax rotation for data from speech audiometry and questionnaires. Bold numbers indicate relevance for a given factor.

	1st Component	2nd Component	3rd Component
SRT for numbers	−0.24	**−0.75**	−0.17
Words 40 dB SPL	0.22	**0.68**	0.05
Words 50 dB SPL	0.47	**0.86**	0.05
Words 65 dB SPL	**0.82**	0.45	0.20
Words 80 dB SPL	**0.88**	0.26	0.25
Sentences in quiet	**0.81**	0.32	0.27
SRT in stationary noise (S0N0)	**−0.79**	−0.41	−0.19
SRT in fluctuating noise (S0NCI)	**−0.81**	−0.22	−0.13
Questionnaire ‘SSQ’	0.14	0.10	**0.98**
Questionnaire ‘listening effort’	−0.21	−0.07	**−0.53**

## Data Availability

Data are provided within the manuscript.
